# Blood and saliva SARS-CoV-2 antibody levels in self-collected dried spot samples

**DOI:** 10.1007/s00430-022-00740-x

**Published:** 2022-06-13

**Authors:** Laura Lahdentausta, Anne Kivimäki, Lotta Oksanen, Marika Tallgren, Sampo Oksanen, Enni Sanmark, Aino Salminen, Ahmed Geneid, Mikko Sairanen, Susanna Paju, Kalle Saksela, Pirkko Pussinen, Milla Pietiäinen

**Affiliations:** 1 Department of Oral and Maxillofacial Diseases, University of Helsinki and Helsinki University Hospital, 00014 Helsinki, Finland; 2 PerkinElmer Finland Oy, 20101 Turku, Finland; 3 Department of Otorhinolaryngology and Phoniatrics–Head and Neck Surgery, Helsinki University Hospital and University of Helsinki, 00029 Helsinki, Finland; 4 Aalto University School of Business, Espoo, Finland; 5 Department of Virology, University of Helsinki and Helsinki University Hospital, 00014 Helsinki, Finland; 6 Institute of Dentistry, University of Eastern Finland, Kuopio, Finland; 7 VTT Technical Research Centre of Finland, 02044 Espoo, Finland

**Keywords:** SARS-CoV-2, Antibody, Blood, Saliva, Dried spot sample, COVID-19, Exposure, Vaccination

## Abstract

We examined the usefulness of dried spot blood and saliva samples in SARS-CoV-2 antibody analyses. We analyzed 1231 self-collected dried spot blood and saliva samples from healthcare workers. Participants filled in a questionnaire on their COVID-19 exposures, infections, and vaccinations. Anti-SARS-CoV-2 IgG, IgA, and IgM levels were determined from both samples using the GSP/DELFIA method. The level of exposure was the strongest determinant of all blood antibody classes and saliva IgG, increasing as follows: (1) no exposure (healthy, non-vaccinated), (2) exposed, (3) former COVID-19 infection, (4) one vaccination, (5) two vaccinations, and (6) vaccination and former infection. While the blood IgG assay had a 99.5% sensitivity and 75.3% specificity to distinguish participants with two vaccinations from all other types of exposure, the corresponding percentages for saliva IgG were 85.3% and 65.7%. Both blood and saliva IgG-seropositivity proportions followed similar trends to the exposures reported in the questionnaires. Self-collected dry blood and saliva spot samples combined with the GSP/DELFIA technique comprise a valuable tool to investigate an individual’s immune response to SARS-CoV-2 exposure or vaccination. Saliva IgG has high potential to monitor vaccination response wane, since the sample is non-invasive and easy to collect.

**Supplementary Information:**

The online version contains supplementary material available at 10.1007/s00430-022-00740-x.

## Introduction

Serological assays are useful in investigating an individual’s immune status and response to vaccinations as well as in perceiving epidemiological information on the spread of the infection. Especially after the implementation of vaccination programs, serological surveys of large populations are essential in evaluating the level and duration of antibody responses [1]. Most serological tests are based on the detection of the IgG and IgM antibodies that recognize SARS-CoV-2 nucleoprotein (N), viral spike glycoprotein S1 subunit, or its receptor-binding domain (RBD), but some applications have also been developed to detect IgA antibodies against these antigens [2–5]. Most SARS-CoV-2 vaccines are designed to use the viral spike glycoprotein or part of it as the immunogen.

Over 90% of subjects start to develop IgG antibodies to SARS-CoV-2 antigens 10–11 days after the onset of symptoms [4, 6, 7]. Serum IgM and IgA levels elevate synchronously to or slightly earlier than IgG, and their seroconversion occurs between days 6 and 15 [8, 9]. A stronger exposure and the severity of the COVID-19 infection are associated with higher antibody levels and a longer durability of antibodies [9, 10].

Mucosal immunity is crucial in limiting respiratory infections. In the oral cavity, class IgG and IgM antibodies mainly diffuse from blood circulation into gingival crevicular fluid and further into the saliva. IgA antibodies are produced in mucosa, and they are responsible for the early humoral immunity against SARS-CoV-2, neutralizing the virus [11, 12]. Serum and saliva anti-SARS-CoV-2 display similar temporal kinetics [8]. Both serum and saliva IgG antibodies are detected up to 9 months after COVID-19 infection, whereas IgA and IgM antibodies decline more rapidly [8, 13]. Significant decrease in serum IgG levels was observed 6 months after second dose of vaccination reflecting the superior long-term humoral response after natural infection compared to vaccine-induced response [14].

Easily performed collection of samples that do not require a laboratory setting is essential for large-scale population screening. Dried blood spot (DBS) samples have been reported to be a valid alternative to plasma/serum collection for anti-SARS-CoV-2 IgG detection as the antibody levels measured from DBS samples correlate with the levels detected from traditional serum/plasma samples [15–18]. Saliva is an easily collectable, non-invasive sample material suitable for anti-SARS-CoV-2 IgG, IgA, and IgM detection [8]. However, dried saliva spot samples have not been utilized in SARS-CoV-2 diagnostics. The aim of our study was to explore the usefulness of self-collected DBS and dried saliva spot (DSS) samples in the analysis of the immune response against SARS-CoV-2. IgG, IgM, and IgA-class antibodies were detected from the dry spot samples collected from 1200 healthcare professionals to find out if DBS and DSS samples can be used to detect an antibody response caused by either natural infection or vaccines. In particular, we were interested to investigate whether saliva is applicable in the antibody analyses.

## Materials and methods

The study population comprised healthcare and social workers, who were recruited between January and March 2021 in the Uusimaa region of Southern Finland. During the COVID-19 epidemic in Finland 78,565 laboratory confirmed cases (1.4% of population) were registered from January 3, 2020 to March 31, 2021 [19]. The worst epidemic situation has been in the southern part of Finland, which has the largest population and highest population density in the country. SARS-CoV-2 vaccinations started in Finland at the beginning of 2021, with critical healthcare workers and risk groups.

The participants worked either in specialized care at the Helsinki University Hospital (HUS) or in primary and social care for the City of Helsinki (HEL). The inclusion criterion for participation was age of at least 18 years. Consent for participation was given using Suomi.fi e-services with a strong identification**.** The participants were recruited through work mailing lists and intranet. In the case of HUS, the participants of the present study were restricted only to those who had participated in the previous questionnaire study concerning COVID-19 exposure among HUS personnel (*n* = 866), presenting a random sample [20]. The list of different occupations among the participants is presented in Supplementary Table 1. The study was conducted according to the guidelines of the Declaration of Helsinki and the study design was approved by the local ethical committees of the Helsinki University Hospital and the City of Helsinki (HUS/1450/2020, HUS/157/2020, HUS/182/2021, HEL 2020-007596T 13 02 01).

Sampling sets for self-collection of blood and saliva samples were delivered to the participants either at their home address or at their workplace. Each sampling set included a PerkinElmer 226 Sample Collection Card designed for dried blood spot (DBS) collection, and equipment and instructions for self-administered saliva and blood sampling. Participants filled in the electronic background questionnaire on their exposures, COVID-19 infections, and vaccinations, and returned the sample card either by mail or to their workplace to be delivered further to the laboratory. Vaccines reported by the participants included Comirnaty (Pfizer-BioNTech), COVID-19 Vaccine AstraZeneca/Vaxzevria (Oxford-AstraZeneca), and Spikevax (Moderna). Altogether, 816 sample cards from HEL and 415 sample cards from HUS were analyzed. 51 sample cards (3.9%) were excluded from further analyses due to insufficient sample material, technical problems, an incompletely filled study number or background questionnaire, or cancelation of study consent by the participant.

### Self-collection of saliva and blood samples

Participants followed detailed written and illustrated instructions for self-collection. Both saliva and blood samplings were advised to be performed before 10 am, and participants were asked not to eat, drink, or brush their teeth for 1 h prior to salivary sampling. Collection cards comprised five equal circles printed in a row and the middle circle was left empty to prevent sample mixing. Two circles printed on the sample collection card were filled with drops of blood drawn from a fingertip with a lancet. Non-stimulated saliva was collected by passive drooling into a plain 15 ml Falcon tube. Drops of saliva were applied on two circles of the collection card using a transfer pipette. After the cards were dried (3–4 h) at room temperature, they were sealed in envelopes and delivered to the laboratory, where they were stored at − 20 °C prior to analysis.

### Antibody analysis

Samples were analyzed at PerkinElmer Wallac Oy, Turku, Finland with a fully automated solid phase DELFIA (time-resolved fluorescence) immunoassay. Punches of 3.2 mm diameter containing approximately 3 μl of blood or saliva were cut from the collection cards into the wells of an assay plate with a DBS Puncher (PerkinElmer Wallac Oy). The sample plate was analyzed with a GSP™ instrument (PerkinElmer Wallac Oy). IgG antibodies against the SARS-CoV-2 spike S1 protein were detected using commercial GSP/DELFIA SARS-CoV-2 IgG kits (PerkinElmer Wallac Oy). IgA and IgM antibodies against the SARS-CoV-2 spike S1 protein were detected using custom-made secondary antibodies and the same kit and GSP protocol as for IgG. The GSP protocol determines fluorescence as counts that are proportional to the amount of human anti-SARS-CoV-2 IgG/IgA/IgM in the sample. Anti-SARS-CoV-2 IgG results were also reported as ratios, which were calculated by dividing the sample signal by the average signal of the calibrator samples provided in the kit. The cut-off value for anti-SARS-CoV-2 IgG in DBS determined by the manufacturer is 1.4. Cut-off values for the other antibody classes and DSS samples have not been determined.

### Comparison of DBS, DSS, and wet saliva samples in pilot sample

Six volunteers with either previous COVID-19 infection or one or two vaccinations collected DBS and DSS samples as described above to test the performance of dried spot saliva samples compared wet saliva samples. In addition, they stored the rest of the non-stimulated saliva (i.e., wet saliva samples) at − 20 °C for further analysis. All samples were collected within 1 week. Anti-SARS-CoV-2 IgG, IgM, and IgA levels were measured from DBS, DSS, and thawed saliva samples with the GSP instrument. For wet saliva analysis, 20 µl of saliva was applied to the wells of a sample plate of GSP/DELFIA SARS-CoV-2 IgG kit (Perkin Elmer Wallac oy) instead of a paper punch. In addition, one volunteer collected a time series of DBS, DSS, and wet saliva samples after receiving the first dose of vaccine.

### Statistical analyses

The antibody levels exhibited a skewed distribution, and they were logarithmically transformed before the statistical analyses. Differences between the groups were analyzed using ANOVA combined with the LSD post hoc test or *t* test. Linear trends were examined using weighted linear terms of ANOVA. Correlations were examined either by Pearson or Spearman analysis, depending on the number of observations. The associations were analyzed using linear regression models using the logarithmically transformed antibody levels as dependent variables, with age, sex, BMI, and smoking as confounding factors, and the level of exposure as independent variables. Each category of the level of exposure was binary coded using the healthy, non-exposed group as the reference. Receiver-operating characteristics (ROC) was used to examine the clinical performance of the assays. Area-under-curve (AUC), and sensitivities and specificities using specified cut-off levels are reported. All statistical analyses were performed with SPSS statistical software (IBM).

## Results

The performance of dry saliva spot (DSS) and wet saliva samples compared to dry blood spot (DBS) samples was tested in a pilot study with six volunteers (Supplementary Fig. 1). The levels of blood anti-SARS-CoV-2 IgG were approximately 10 to even 100 times higher than those of DSS and wet saliva samples, while the differences were more moderate in IgM and IgA. IgG (*r* = 0.71) and IgM (*r* = 0.94) of dry and wet saliva samples exhibited a strong correlation (Spearman’s rho), whereas IgA (*r* = − 0.086) did not. The levels of blood IgG, IgM, and IgA started to increase 7 days after the vaccination. Saliva IgG levels increased only moderately after vaccination, whereas IgM and IgA counts remained at the baseline level (Supplementary Fig. 2).

A total of 1231 persons participated in the study; 816 (66.3%) worked in HEL and 415 (33.7%) in HUS. Their characteristics are presented in Table 1. Mean age did not differ significantly between the groups, but the HUS population included more middle-aged (40–59 years) participants. The gender distribution was similar in both groups with approximately 10% male participants. The occupational groups differed between the populations; this was one of the reasons to include both city and hospital district workers in the study. In total, nurses were the largest group (60.7%), followed by physicians (9.0%), therapists (8.9%), social workers (7.8%), dental care professionals (7.5%), and those in administrative or maintenance work (6.2%). Different occupations under each of the six groups are listed in Supplementary table 1.

**Table 1:** Characteristics of the population

	Whole population, *n* = 1231	HEL, *n* = 816	HUS, *n* = 415	*p* value^1^
	Mean (SD)			
Age, years	43.8 (11.3)	43.5 (11.6)	44.3 (10.5)	0.197
BMI, kg/m^2^	26.7 (5.84)	26.8 (6.00)	26.6 (5.54)	0.644
	*N* (%)			*p* value^2^
Age groups				
18–29 years	150 (12.2)	114 (14.0)	36 (8.7)	**0.012**
30–39 years	333 (27.2)	222 (27.3)	111 (26.8)	
40–49 years	299 (24.2)	189 (23.3)	110 (26.6)	
50–59 years	339 (27.7)	210 (25.9)	129 (31.2)	
60-years	105 (8.6)	77 (9.5)	28 (6.8)	
Sex				
Males	108 (9.6)	73 (10.3)	35 (8.4)	0.330
Females	1016 (90.2)	636 (89.5)	380 (91.6)	
Smoking				
Never	768 (70.1)	482 (67.8)	286 (74.3)	**0.025**
Ever	328 (29.9)	229 (32.2)	99 (25.7)	
BMI				
Normal	505 (46.2)	339 (47.7)	166 (43.3)	0.186
Overweight	331 (30.3)	202 (28.5)	129 (33.7)	
Obese	257 (23.5)	169 (23.8)	88 (23.0)	
Occupation				
Physicians	111 (9.0)	47 (5.8)	64 (15.4)	** < 0.001**
Nurses	746 (60.7)	396 (48.6)	350 (84.3)	
Dental	92 (7.5)	92 (11.3)	0	
Social worker or psychologist	96 (7.8)	96 (11.8)	0	
Therapist	109 (8.9)	109 (13.4)	0	
Administration or maintenance	76 (6.2)	75 (9.2)	1 (0.2)	
Antigen exposure				
Healthy	350 (31.3)	262 (36.8)	88 (21.2)	** < 0.001**
Exposed	381 (33.8)	184 (25.9)	197 (47.5)	
Vaccinated once	115 (10.2)	91 (12.8)	24 (5.8)	
Vaccinated twice	204 (18.1)	125 (17.6)	79 (19.0)	
Former COVID-19 infection	57 (5.1)	34 (4.8)	23 (5.5)	
Former COVID-19 infection and vaccinated once or twice	19 (1.7)	15 (2.1)	4 (1.0)	

Statistically significant p values *p* < 0.05 are presented as bolded values

^1^*t* test

^2^Chi-square test; differences between HEL and HUS

**Fig. 1:**
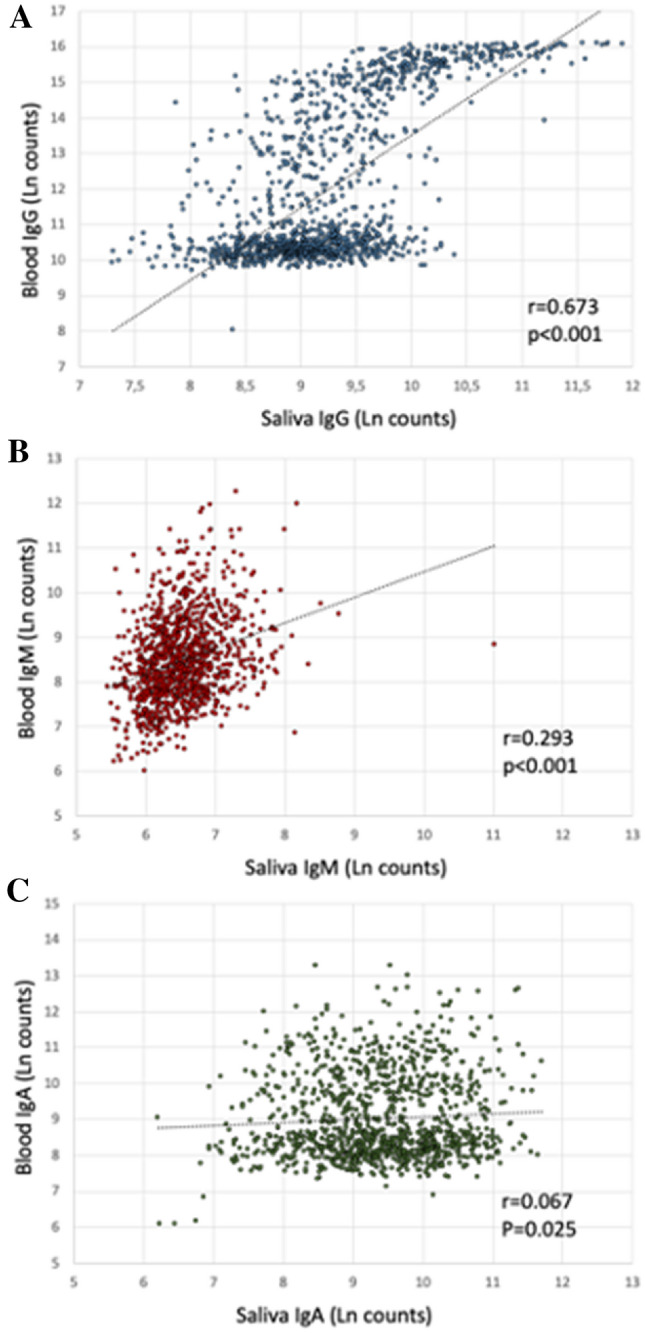
Correlation between saliva and blood anti-SARS-CoV-2 antibody levels. The logarithmically transformed antibody counts are presented. Each figure shows one antibody class with corresponding antibodies measured from blood (DBS) and saliva (DSS). The correlation coefficient (*r*) and *p* value from Pearson correlation analyses are shown. A number of participants are: IgG, *n* = 1186; IgM, *n* = 1077; IgA, *n* = 1079

In the study group of health care workers, IgG and IgM of DBS and DSS samples correlated relatively well with *r* = 0.673 (*p* < 0.001) and *r* = 0.293 (*p* < 0.001), respectively, whereas IgA in DBS and DSS samples correlated only weakly (*r* = 0.067, *p* = 0.025) (Fig. 1). The antibody levels of the study group are presented in Supplementary Table 2.

**Fig. 2:**
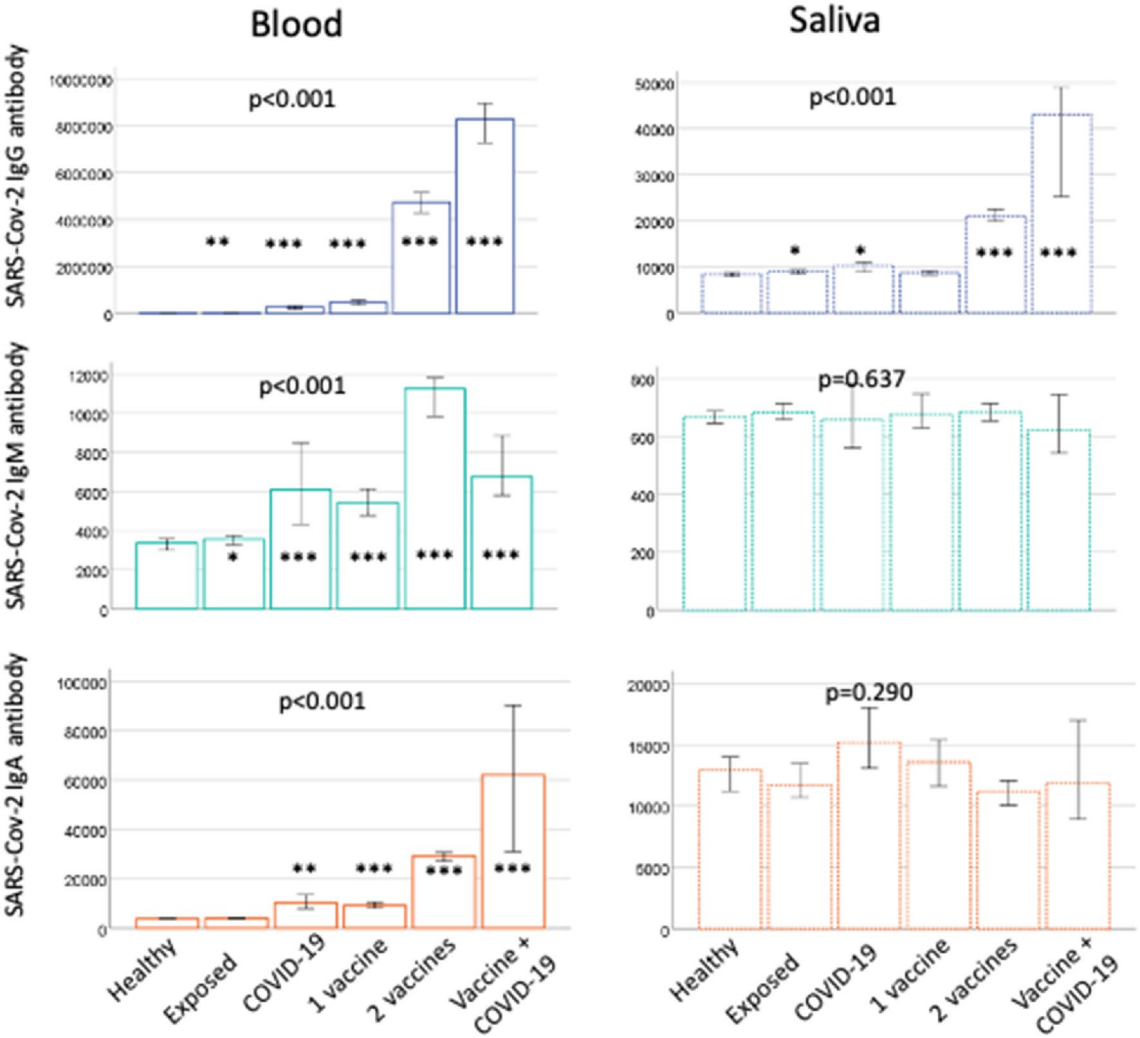
SARS-Cov-2 antibody levels in the groups with various levels of exposure. IgG-, IgM-, and IgA-class antibody levels were determined from the dried spot blood and saliva samples. Median levels with IQR are shown. The groups are: (1) healthy = non-infected, non-exposed without vaccination; (2) exposed = registered as ‘negative qPCR result’, ‘quarantine due to exposure’ or ‘exposure in the family’; (3) former COVID-19 infection (4) vaccinated once; (5) vaccinated twice; (6) former COVID-19 infection and vaccinated once or twice. The levels were logarithmically transformed for statistical testing. The *p* values above are from the ANOVA test, significance of the differences between the groups. The stars depict the level of significance compared to the group of healthy as produced by LSD, *p < 0.05; **p < 0.01; ***p < 0.001

The exposure level was determined according to the questionnaire data collected from participants and divided into the following groups: (1) healthy = non-infected, non-exposed, non-vaccinated (*n* = 350, 31.0%); (2) exposed = registered as ‘negative qPCR result’, ‘quarantine due to exposure’, or ‘exposure in the family’, non-vaccinated (*n* = 381, 33.9%); (3) former COVID-19 infection (*n* = 57, 5.1%); (4) vaccinated once (*n* = 115, 10.2%); (5) vaccinated twice (*n* = 204, 18.1%); and (6) former COVID-19 infection and vaccinated once or twice (*n* = 19, 1.7%). The characteristics did not differ between the exposure groups (Supplementary Table 3). The median antibody concentrations in different exposure groups are presented in Fig. 2. Blood antibody levels and saliva IgG displayed significant (*p* < 0.001) increasing trends among the participants with different exposure level, whereas saliva IgM and IgA did not. Blood IgG and IgM as well as saliva IgG levels were significantly higher in all exposure groups compared to “healthy” participants. Compared to “healthy”, blood IgA levels were significantly higher in all other groups than “exposed”. The associations of the antibody levels with different levels of exposure were examined using linear regression models (Table 2). For blood IgG (*R*^2^ = 0.146), IgM (*R*^2^ = 0.0.65), and IgA (*R*^2^ = 0.014), as well as saliva IgG (*R*^2^ = 0.0.57), the exposure level was the main determinant of the antibody concentration. Age was inversely associated with blood IgG and IgM as well as saliva IgM and IgA, whereas sex was not associated with any of the antibody concentrations.

**Table 2:** Associations of the antibodies with the level of exposure

	Antibody concentration^1^					
	Blood IgG	Blood IgM	Blood IgA	Saliva IgG	Saliva IgM	Saliva IgA
	β, *p* value					
Covariates^2^						
Age, years	**− 0.069, 0.023**	**− 0.071, 0.026**	–	–	**− 0.066, 0.044**	**− 0.176, < 0.001**
Sex^3^	–	–	–	–	–	–
BMI, kg/m^2^	–	–	–	**0.074, 0.017**	–	–
Smoking^4^		–	–	–	**− 0.069, 0.036**	–
Level of exposure^5^						
Exposed	**0.118, 0.002**	0.066, 0.096	− 0.065, 0.103	**0.100, 0.009**	0.013, 0.747	− 0.027, 0.500
Former COVID-19 infection	**0.434, < 0.001**	**0.284, < 0.001**	**0.213, < 0.001**	**0.132, 0.010**	0.005, 0.923	0.054, 0.318
1 vaccination	**0.530, < 0.001**	**0.291, < 0.001**	**0.276, < 0.001**	**0.101, 0.036**	0.060, 0.239	0.029, 0.561
Former COVID-19 infection and vaccination	**0.625, < 0.001**	**0.231, < 0.001**	**0.343, < 0.001**	**0.461, < 0.001**	− 0.027, 0.634	− 0.035, 0.537
2 vaccinations	**0.883, < 0.001**	**0.556, < 0.001**	**0.398, < 0.001**	**0.590,** **< 0.001**	0.019, 0.689	− 0.081, 0.078

Statistically significant p values *p* < 0.05 are presented as bolded values

^1^Logarithmically transformed (natural logarithm)

^2^Only statistically significant estimates are shown

^3^1 = man, 2 = woman

^4^0 = never, 1 = ever

^5^Binary coding of each category; healthy = non-infected, non-exposed, non-vaccinated as the reference; adjusted for standardized predicted value of the covariates

**Fig. 3:**
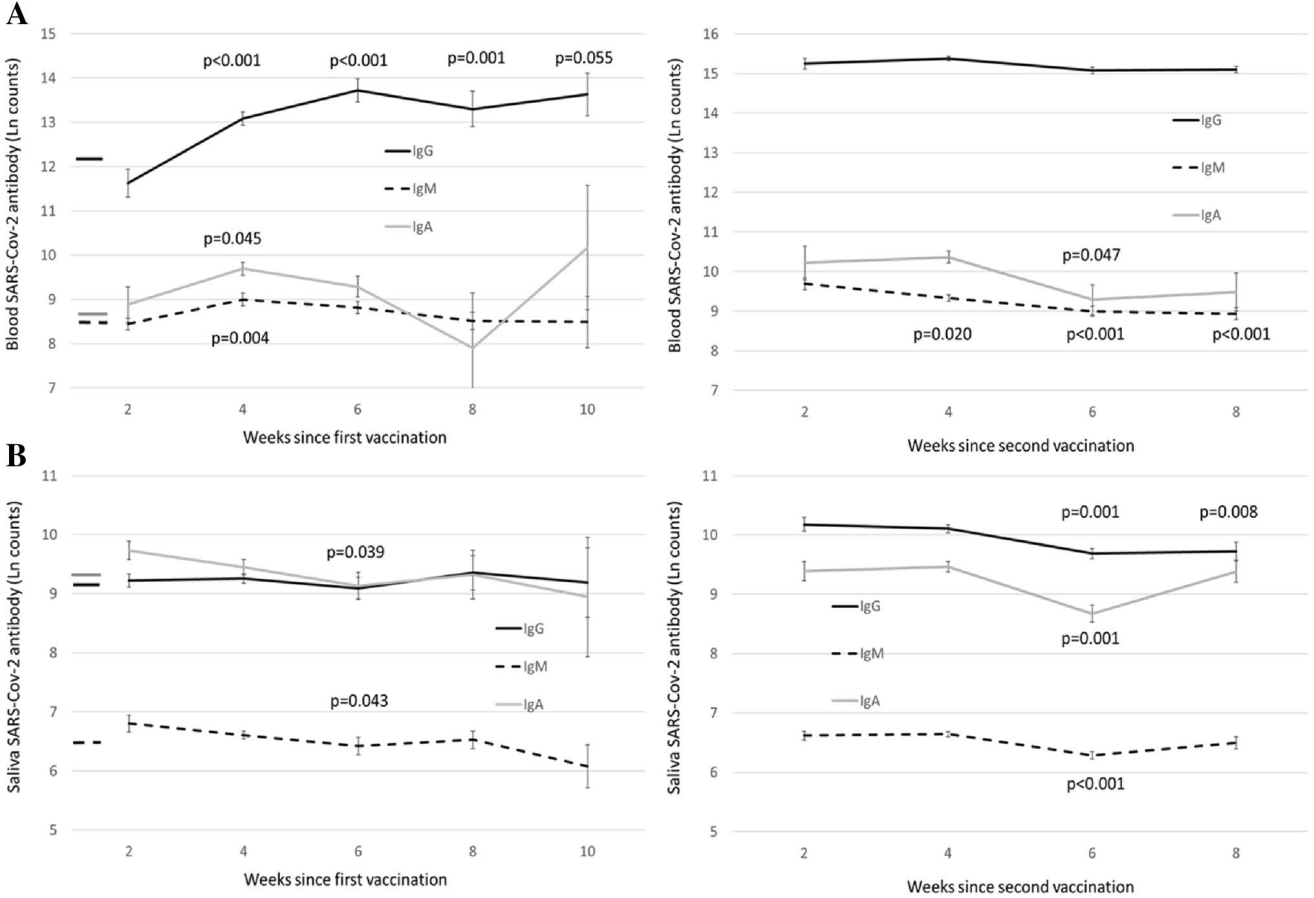
Effect of time after vaccination on the antibody levels. **A** Blood antibody levels and **B** saliva antibody levels. Mean values with SE are shown and the *p* values are produced by the LSD test. Lines on the left side represent the mean values of healthy, unexposed participants for reference

In participants with former COVID-19 infection, the median time from diagnosis to sampling was 38.6 weeks (IQR 32.6, range 4.0–54.4 weeks) (Supplementary Fig. 3). None of the antibody levels had a significant correlation with the time since infection (data not shown). Next, we analyzed the effect of time since vaccination on antibody levels (Fig. 3). Blood IgG levels increased with a significant linear trend (*p* < 0.001) until 10 weeks after the first vaccination, whereas no significant changes were observed after the second vaccination. No significant trends were observed in blood IgM and IgA levels after the first vaccination, whereas they both exhibited linear decreasing trends after the second vaccination (< 0.001 and 0.020). Saliva IgA (*p* = 0.031) and IgM (*p* = 0.025) decreased linearly after the first vaccination, and all saliva antibody levels decreased after the second vaccination (*p* for weighted trend: IgG < 0.001, IgM 0.005, and IgA 0.041).

**Fig. 4:**
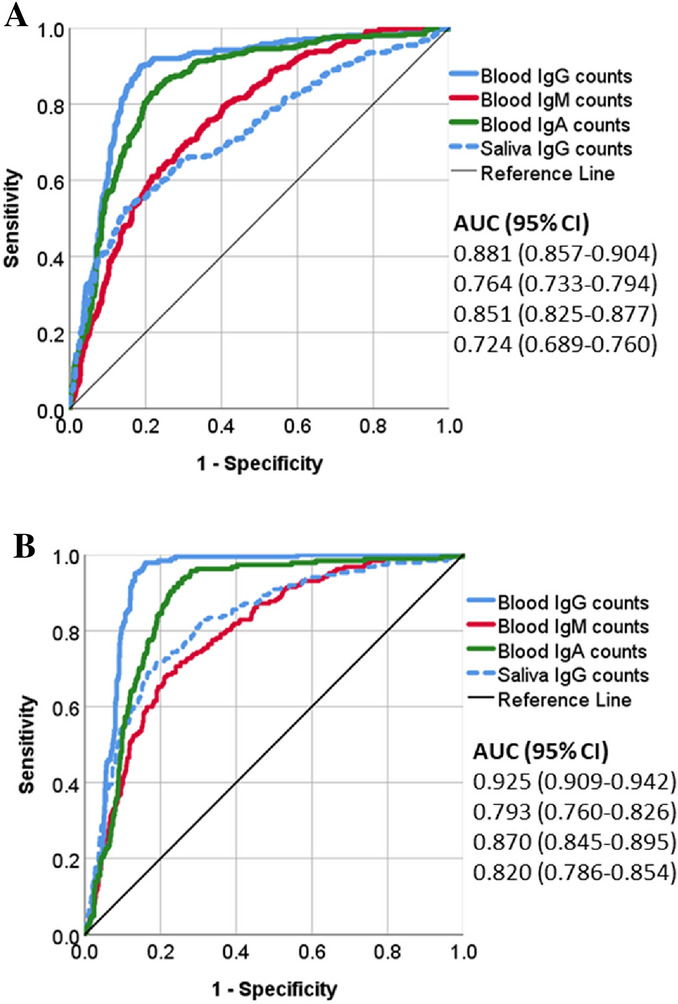
Performance of DBS and DSS determinations to detect vaccinated participants. ROC-analyses were performed for DBS-IgG, IgM, and IgA, and DSS-IgG. The comparisons were made between **A** participants who were vaccinated vs. others, and **B** participants who were vaccinated twice vs. others

The performance of the assays to distinguish the exposure groups was further investigated using ROC analyses (Supplementary Table 4). All blood antibody assays, and saliva IgG differentiated successfully participants with vaccination and/or former COVID-19 infection from healthy (*p* < 0.001). These assays also presented highly significant (*p* < 0.001) AUC values, when participants with vaccinations were detected among the entire study group with different levels of exposure (Fig. 4).

Using the seropositivity cut-off value of the blood IgG ratio defined by the manufacturer, we determined the cut-off level for saliva IgG: the best performance of the saliva assay (DSS) was obtained with a saliva IgG ratio of 0.14, resulting in a 70.0% sensitivity and 75.5% specificity in detecting seropositive participants. The true-positive rates of DBS and DSS seropositivities were calculated to detect participants with former COVID-19 infection, vaccination, and two doses of vaccines (Table 3). The DBS assay had a 99.5% sensitivity and 75.3% specificity in finding participants with two vaccinations, and the corresponding percentages for DSS were 85.3% and 65.7%.

**Table 3:** Calculation of true-positive rates for DBS- and DSS-IgG seropositive values

	Infected	Not infected		Infected	Not infected
DBS seropositive^1^	43	108	DSS seropositive	23	230
DBS seronegative^1^	14	623	DSS seronegative	32	466
Sensitivity	75.4%			41.8%	
Specificity	85.2%			67.0%	

	Vaccinated	Not vaccinated		Vaccinated	Not vaccinated
DBS seropositive^2^	308	108	DSS seropositive	221	230
DBS seronegative^2^	30	466	DSS seronegative	109	466
Sensitivity	91.1%			67.0%	
Specificity	81.2%			67.0%	

	Vaccinated twice	Not vaccinated twice		Vaccinated twice	Not vaccinated twice
DBS seropositive^2^	202	214	DSS seropositive	168	283
DBS seronegative^2^	1	652	DSS seronegative	29	546
Sensitivity	99.5%			85.3%	
Specificity	75.3%			65.7%	

Statistically significant p values *p* < 0.05 are presented as bolded values

^1^Groups healthy, exposed, and infected; *n* = 788

^2^Groups healthy, exposed, and vaccinated; *n* = 1069

**Fig. 5:**
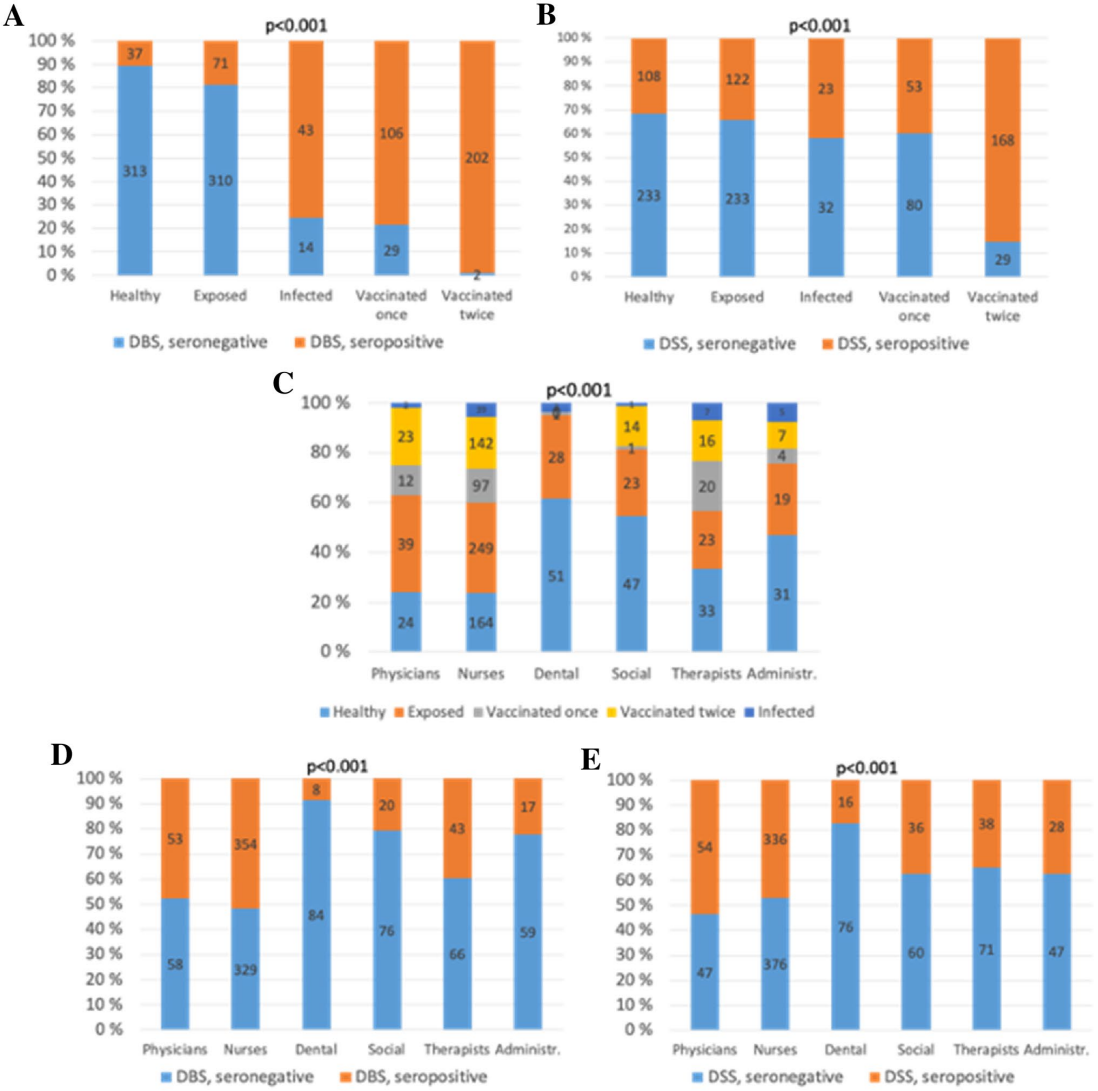
Reported exposures and seropositivity in DBS and DSS measurements. Exposure is defined as healthy, exposed, vaccinated once or twice, and confirmed COVID-19 infection. DBS-seropositivity is determined as blood IgG ratio exceeding the cut-off value of 1.4. DSS-seropositivity is determined as saliva IgG ratio exceeding the cut-off value of 0.14. The proportion of seropositivity in DBS and DSS determinations is presented in **A**–**B** different exposure groups, and **D**–**E** different occupational groups. The *p* value is produced by Chi-square test

The proportion of DBS-seropositive subjects increased (*p* < 0.001) among healthy, exposed, infected, vaccinated once, and vaccinated twice as follows: 10.6%, 18.6%, 75.4%, 76.3%, and 99.0% (Fig. 5A). The corresponding proportions for DSS-seropositive subjects were 31.7%, 34.4%, 41.8%, 39.8%, and 85.3% (Fig. 5B). The exposure differed between occupational groups according to both the questionnaire (Fig. 5C) and the proportions of seropositivities (Fig. 5D and E).

## Discussion

We demonstrated that self-administered dry spot blood (DBS) and saliva (DSS) samples analyzed with the GSP/DELFIA system can be utilized in analyzing individual immune responses to SARS-CoV-2. All three antibody classes in DBS samples, IgG, IgM and IgA, and IgG in DSS samples were able to distinguish infected and/or vaccinated individuals from the healthy, non-exposed, non-vaccinated group. Importantly, all blood assays and saliva IgG identified vaccinated participants from the whole population: using the DBS-IgG-seropositivity cut-off level determined by the manufacturer, the assay had a sensitivity of 99.5% in differentiating twice vaccinated participants, whereas the corresponding cut-off level for DSS-IgG determined in this study had an 85.3% sensitivity. Thus, the DBS samples would be highly useful in following immune responses after SARS-CoV-2 vaccination. Since saliva collection is easy and non-invasive, DSS also offers good potential in the follow-up of immune responses to determine, e.g., needs for additional doses of vaccinations.

All antibody classes could be measured from DBS samples. IgG class antibodies comprise 70–75% of all immunoglobulins found in the blood, and as expected, SARS-CoV-2 IgG analyses gave the highest counts in the analyses. In addition, IgA (10–15% of all Igs) and IgM (5% of all Igs) could be measured from DBS samples. IgM and IgA usually disappear from the circulation with viral clearance and are not recommended in the follow-up after infection, but they may help to elucidate the clinical picture in the patients [21]. Performance of a serological assay may improve if different immunoglobulin classes are combined [5]. Thus, further studies using DBS samples are warranted among acute or convalescent phase patients.

Saliva contains IgG and IgM class antibodies diffused from blood and secretory IgA produced on the oral mucosa. In general, the measured antibody levels in saliva are approximately 10–100 times lower than in the blood [22, 23], which was also shown in our pilot sample comparing wet and dry saliva samples to dry blood samples. Low antibody levels in saliva creates a challenge with DDS samples, since only a small sample volume is used in the measurements. Nevertheless, our results showed relatively good correlation between blood and saliva anti-SARS-CoV-2 IgG levels. In addition, saliva IgM levels correlated significantly with blood IgM levels, but correlation between blood and saliva IgA levels was only modest. This most likely reflects the different origin of blood and saliva IgA. The usefulness of DSS-IgA was not fully clarified here due to the study design, but its performance would be interesting to test among acute phase COVID-19 patients or subjects who received oral/mucosal vaccines.

Only 1% of twice vaccinated participants remained IgG seronegative in the analyses, whereas 25% of infected persons were seronegative. This phenomenon may be explained by the long time between infection and sample collection: the median time was 39 weeks, but ranged between 4 and 54 weeks. Typically, a good immune response against SARS-CoV-2 spike protein is seen 10–14 days after the onset [9], but there are also individuals who do not seroconvert after SARS-CoV-2 infection [22, 23]. As neutralizing antibody responses and specific memory B cells have been described as remaining in the circulation for up to ≥ 8 months (32 weeks), previously infected individuals may harbor antibodies at this time point [21]. The immune response, however, declines at the latest 12 months (48 weeks) after infection, and after 10 months (40 weeks) post-infection 13% of individuals lost detectable IgG titers [24, 25]. We did not observe a declining trend of the antibody levels after COVID-19, and the seronegative subjects were also distributed randomly along the time axis. At the beginning of the COVID-19 pandemic, qPCR tests were restricted to patients admitted to hospital, and therefore not all assumed SARS-CoV-2 infections were qPCR verified. However, the antibody levels did not correlate with the time since infection even if only the infected participants with a positive qPCR test result were included. Thus, the sensitivity of the DBS-seronegative results in identifying participants with formed infection was only 75.4%, whereas the specificity of 85.2% was acceptable.

IgG levels measured from DSS samples distinguished individuals with one or two doses of vaccines from the healthy, unexposed, and unvaccinated participants with significant AUCs, but importantly, the assay provided good and excellent performance in distinguishing vaccinated persons from the whole population (AUC 0.72 and 0.82, respectively). In the present study, we had a chance to perform follow-up only up to 8 weeks since the second dose of vaccination, and longer monitoring will be feasible. mRNA vaccine-induced antibodies have been detected more than 6 months after vaccination [26]. Nevertheless, measuring the antibodies from easily collectable saliva samples to estimate the optimal time for a booster vaccination would save health care costs and prevent later breakthrough infections.

Measured SARS-CoV-2 antibodies correlated well with the level of exposure. As expected, two dosages of vaccine and natural infection combined with vaccination induced the highest antibody levels. With the BioNTech Pfizer mRNA vaccine, it has been reported that the antibody, especially IgA, levels were higher in individuals who had a positive COVID-19 history compared to those with a negative one [27, 28]. This reflects the secondary immune response and was also observed in the present study. Healthcare workers have been at higher risk for COVID-19 infection than the general population [29]. In our study, seropositivity was most frequent among nurses, physicians, and therapists, indicating that work-related transmission may have occurred to some extent. At the beginning of the COVID-19 epidemic, the Finnish government declared a state of emergency that continued until June 2020. During that time, a large proportion of non-urgent care was postponed, and remote patient contact was preferred. Especially in dentistry, only acute dental care was given at that time in the southern part of Finland.

Studying the effects of recent exposures to COVID-19 was not possible due to the study design. Thus, it remains to be investigated whether natural infection is reflected short term in the saliva IgA and IgM levels. We recruited the participants through work mailing lists and the occupational groups could not be selected beforehand. Therefore, the group sizes differ and relatively few participants representing dental professionals, social workers, and administrative staff could be recruited, which may bias the results. Additionally, in this study, we do not have samples collected at different time points from the same person, and thus, we can estimate the long-term antibody response only at the group level. In Finland, the second dose of SARS-CoV-2 vaccination was first given 3 weeks after the first dose. The vaccination policy changed quite soon, and the second dose was given 3 months after the first dose. The changes in vaccination policy may be reflected in the results. The strength of our study is a relatively large sample size containing participants with different exposure levels: our data include information from healthy, exposed, infected, and once or twice vaccinated participants, enabling the comparison of antibody responses in different groups.

## Conclusions

Our results indicate that self-collected dried blood and saliva spot samples can be used reliably in SARS-CoV-2 antibody analyses to measure the immune response to SARS-CoV-2 vaccination and monitor waning humoral immune response after vaccinations and natural infection. Both blood and saliva assays displayed excellent accuracy in differentiating high IgG levels after two doses of vaccination. Dried spot samples are easily collected at home, thus enabling large sample collection without requiring specialized personnel for sample taking.

## Supplementary Information

Below is the link to the electronic supplementary material.

Supplementary file1 (DOCX 5193 KB)
