# Supplementary material for: Blood and saliva SARS-CoV-2 antibody levels in self-collected dried spot samples
Source: Med Microbiol Immunol. 2022 Jun 13;211(4):173–83. doi: 10.1007/s00430-022-00740-x (PMC9191541; doi:10.1007/s00430-022-00740-x)
Supplement: Supplementary file 1 — Supplementary file1 (DOCX 5193 KB) [file 430_2022_740_MOESM1_ESM.docx]

**Supplementary table 1. The list of different occupations among the participants.**

| **Occupation group** | Occupation |
| --- | --- |
| **Nurses** |  |
|  | Registered nurse |
|  | Public health nurse |
|  | Practical nurse |
|  | Paramedic |
|  | Midwife |
|  | Laboratory assistant |
|  | Radiology nurse |
|  | Head nurse |
|  | Hygiene nurse |
|  | Disability nurse |
|  | District nurse |
| **Physicians** | Physician |
| **Therapists** |  |
|  | Psychologist |
|  | Occupational therapist |
|  | Speech therapist |
|  | Mental health nurse |
|  | Social therapist |
|  | Rehabilitation assistant |
|  | Physical education instructor |
| **Social workers** |  |
|  | Social counsellor |
|  | Social worker |
|  | Counsellor |
|  | Cultural counsellor |
|  | Child welfare worker |
| **Dental care professionals** |  |
|  | Dentist |
|  | Dental assistant |
|  | Dental hygienist |
| **Administrative or maintenance work** |  |
|  | Service chief |
|  | Administrative assistant |
|  | Customer servant |
|  | Technician |
|  | Institutional cleaners |
|  | Ward clerk |
|  | Deputy director |
|  | Janitor |
|  | Archivist |

**Supplementary table 2. Antibody levels in the population**

|  |  | Whole population, n=1231 | HEL,  n=816 | HUS,  n=415 |  |
| --- | --- | --- | --- | --- | --- |
|  |  | Mean (SD) | | | p-value^2^ |
| Blood ^1^ | IgG counts | 12.1 (2.20) | 11.8 (2.08) | 12.5 (2.37) | <0.001 |
|  | IgG ratio | 0.75 (2.20) | 0.51 (2.08) | 1.23 (2.37) | <0.001 |
|  | IgM counts | 8.52 (0.97) | 8.48 (0.97) | 8.58 (0.98) | 0.094 |
|  | IgA counts | 8.58 (2.43) | 8.94 (1.16) | 7.98 (3.22) | <0.001 |
| Saliva ^1^ | IgG counts | 9.29 (0.72) | 9.24 (0.74) | 9.39 (0.67) | 0.001 |
|  | IgG ratio | -2.02 (0.71) | -2.09 (0.72) | -1.86 (0.66) | <0.001 |
|  | IgM counts | 6.56 (0.49) | 6.58 (0.47) | 6.51 (0.53) | 0.054 |
|  | IgA counts | 9.38 (0.94) | 9.46 (0.93) | 9.24 (0.05) | <0.001 |

^1^ Logarithmically transformed levels (LN)

^2^ t-test between HEL and HUS

|  |  | Whole population, n=1231 | HEL,  n=816 | HUS,  n=415 |  |
| --- | --- | --- | --- | --- | --- |
|  |  | Median (IQR) | | | p-value^1^ |
| Blood | IgG counts | 43279 (1761007) | 40842 (645343) | 62626 (3436925) | 0.001 |
|  | IgG ratio | 0.54 (22.3) | 0.49 (7.71) | 0.84 (46.8) | <0.001 |
|  | IgM counts | 4510 (6838) | 4280 (5914) | 4952 (7921) | 0.030 |
|  | IgA counts | 5204 (16182) | 4948 (13337) | 7164 (22261) | 0.016 |
| Saliva | IgG counts | 9870 (9137) | 9251 (8128) | 11108 (10067) | <0.001 |
|  | IgG ratio | 0.12 (0.12) | 0.11 (0.10) | 0.15 (0.14) | <0.001 |
|  | IgM counts | 673 (421) | 688 (427) | 649 (411) | 0.011 |
|  | IgA counts | 12219 (17588) | 13311 (18935) | 10583 (15313) | <0.001 |

^1^ Mann-Whitney test

**Supplementary table 3. Characteristics of the population stratified by exposure level.**

|  |  | **Healthy, n=350** | **Exposed, n=378** | **Vaccinated once, n=57** | **Vaccinated twice, n=115** | **Former COVID-19 infection, n=203** | **Former COVID-19 infection and vaccinated once or twice n=19** |  |
| --- | --- | --- | --- | --- | --- | --- | --- | --- |
|  |  | **Mean (SD)** | | | | | | **p-value^1^** |
| **Age** | Years | 43.8 (11.3) | 43.6 (10.6) | 43.9 (11.8) | 43.8 (11.6) | 43.8 (11.6) | 46.8 (11.2) | 0.970 |
| **BMI** | kg/m^2^ | 26.4 (6.2) | 26.9 (5.8) | 26.7 (6.9) | 26.4 (6.1) | 26.8 (4.6) | 28.4 (6.6) | 0.705 |
|  |  | **N (%)** | | | | | | **p-value^2^** |
| **Age groups (years)** | 18-29 | 42 (12.0) | 38 (10.1) | 8 (14.0) | 15 (13.0) | 31 (15.3) | 3 (15.8) | 0.750 |
|  | 30-39 | 97 (27.7) | 104 (27.5) | 15 (26.3) | 33 (28.7) | 48 (23.6) | 3 (15.8) |  |
|  | 40-49 | 83 (23.7) | 104 (27.5) | 13 (22.8) | 22 (19.1) | 51 (25.1) | 3 (15.8) |  |
|  | 50-59 | 99 (28.3) | 108 (28.6) | 16 (28.1) | 30 (26.1) | 56 (27.6) | 8 (42.1) |  |
|  | 60- | 29 (8.3) | 24 (6.3) | 5 (8.8) | 15 (13.0) | 17 (8.4) | 2 (10.5) |  |
| **Sex** | Males | 38 (10.9) | 32 (8.4) | 2 (3.5) | 6 (5.2) | 28 (13.8) | 2 (10.5) | 0.065 |
|  | Females | 311 (89.1) | 349 (91.6) | 55 (96.5) | 109 (94.8) | 175 (86.2) | 17 (89.5) |  |
| **Smoking** | Never | 252 (75.9) | 253 (66.4) | 39 (70.9) | 81 (71.7) | 130 (66.3) | 13 (68.4) | 0.097 |
|  | Ever | 80 (24.1) | 128 (33.6) | 16 (29.1) | 32 (28.3) | 66 (33.7) | 6 (31.6) |  |

^1^ one-way ANOVA; ^2^ Chi-square test

**Supplementary table 4. Receiver operating characteristics (ROC) to distinguish different levels of exposure.**

| **Sample** | **Antibody** | **Area under curve** | **95% CI** | **p-value** |
| --- | --- | --- | --- | --- |
| **A) Vaccinated twice vs. healthy** | | | | |
| Blood | IgG | 0.959 | 0.941-0.977 | **<0.001** |
|  | IgM | 0.842 | 0.807-0.877 | **<0.001** |
|  | IgA | 0.926 | 0.899-0.953 | **<0.001** |
| Saliva | IgG | 0.851 | 0.816-0.887 | **<0.001** |
|  | IgM | 0.513 | 0.461-0.566 | 0.620 |
|  | IgA | 0.461 | 0.408-0.514 | 0.147 |
| **B) Vaccinated once vs. healthy** | | | | |
| Blood | IgG | 0.835 | 0.789-0.880 | **<0.001** |
|  | IgM | 0.717 | 0.667-0.768 | **<0.001** |
|  | IgA | 0.825 | 0.782-0.869 | **<0.001** |
| Saliva | IgG | 0.588 | 0.527-0.649 | **0.004** |
|  | IgM | 0.521 | 0.460-0.581 | 0.502 |
|  | IgA | 0.508 | 0.447-0.570 | 0.787 |
| **C) Former infection vs. healthy** | | | | |
| Blood | IgG | 0.846 | 0.790-0.902 | **<0.001** |
|  | IgM | 0.708 | 0.630-0.785 | **<0.001** |
|  | IgA | 0.808 | 0.740-0.876 | **<0.001** |
| Saliva | IgG | 0.569 | 0.487-0.652 | 0.119 |
|  | IgM | 0.512 | 0.422-0.603 | 0.782 |
|  | IgA | 0.558 | 0.477-0.638 | 0.195 |
| **D) Exposed vs. healthy** | | | | |
| Blood | IgG | 0.537 | 0.492-0.582 | 0.110 |
|  | IgM | 0.523 | 0.478-0.568 | 0.317 |
|  | IgA | 0.557 | 0.512-0.602 | **0.015** |
| Saliva | IgG | 0.545 | 0.500-0.590 | 0.053 |
|  | IgM | 0.513 | 0.467-0.558 | 0.582 |
|  | IgA | 0.496 | 0.451-0.542 | 0.878 |

**
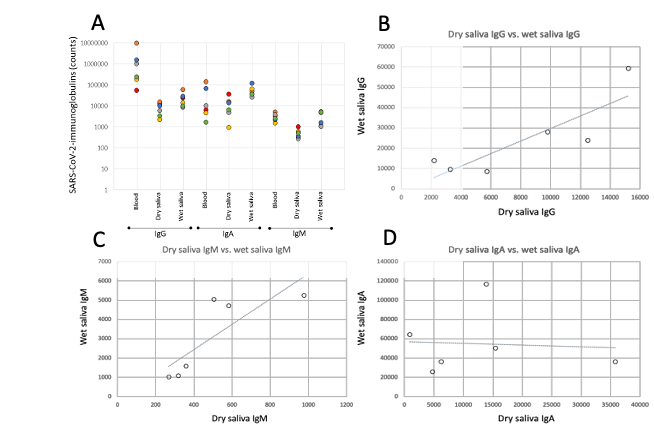
**

**Supplementary figure 1. Comparison of DBS, DSS, and wet saliva as sample materials for SARS-CoV-2 antibody detection.** A) Samples were collected from six volunteers and the results are presented individually. Different immunoglobulin classes of dry and wet saliva samples are presented in separately: B) IgG, C) IgM, and D) IgA.


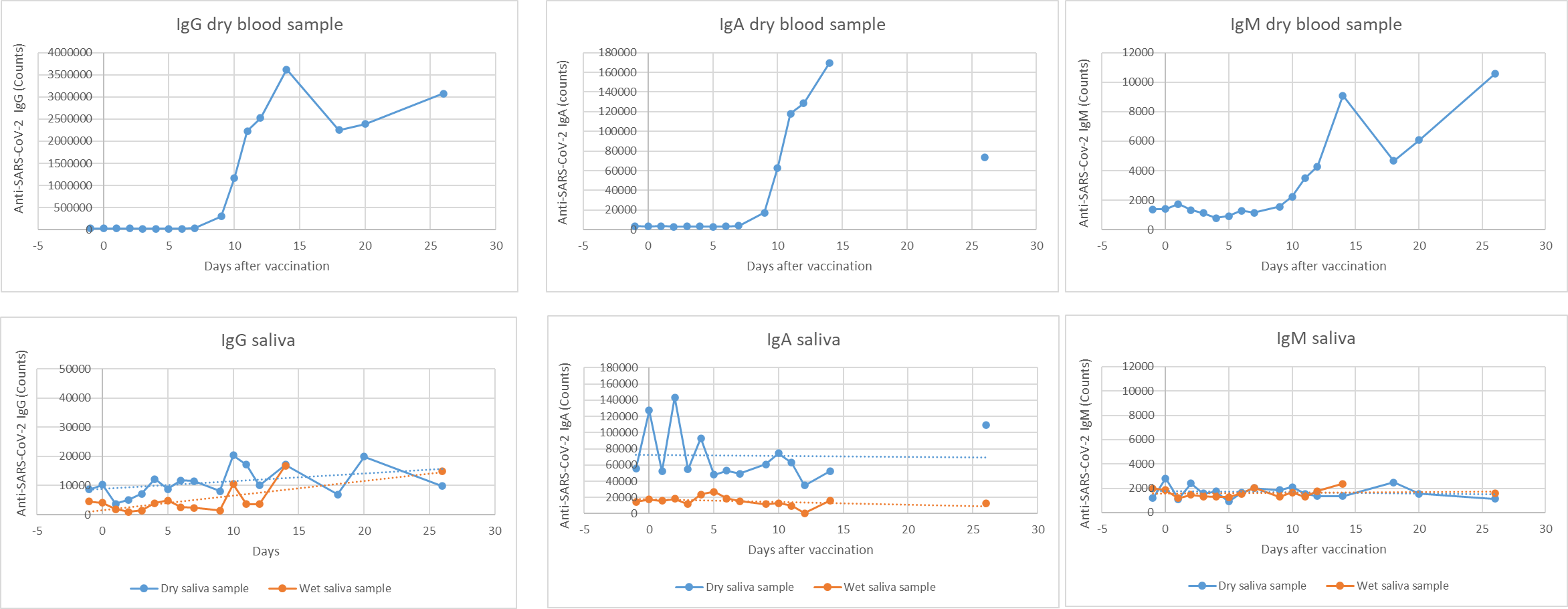


**Supplementary figure 2**. **Antibody response of one person to the first vaccine dosage against SARS-CoV-2.** The antibodies were measured from DBS, DSS, and wet saliva samples. Vaccine (Comirnaty, Pfizer-BioNTech) was given on day zero.


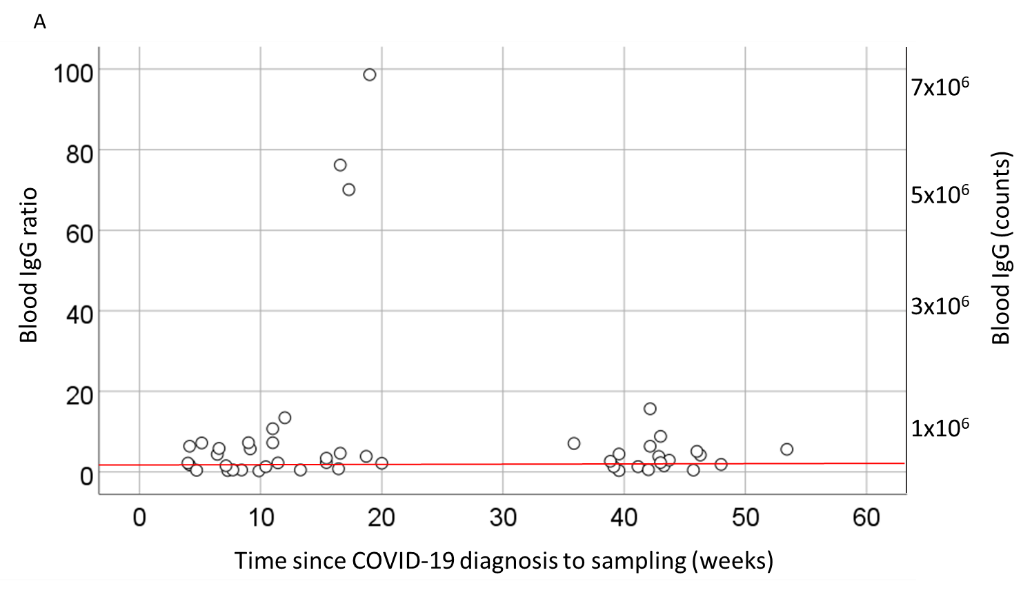


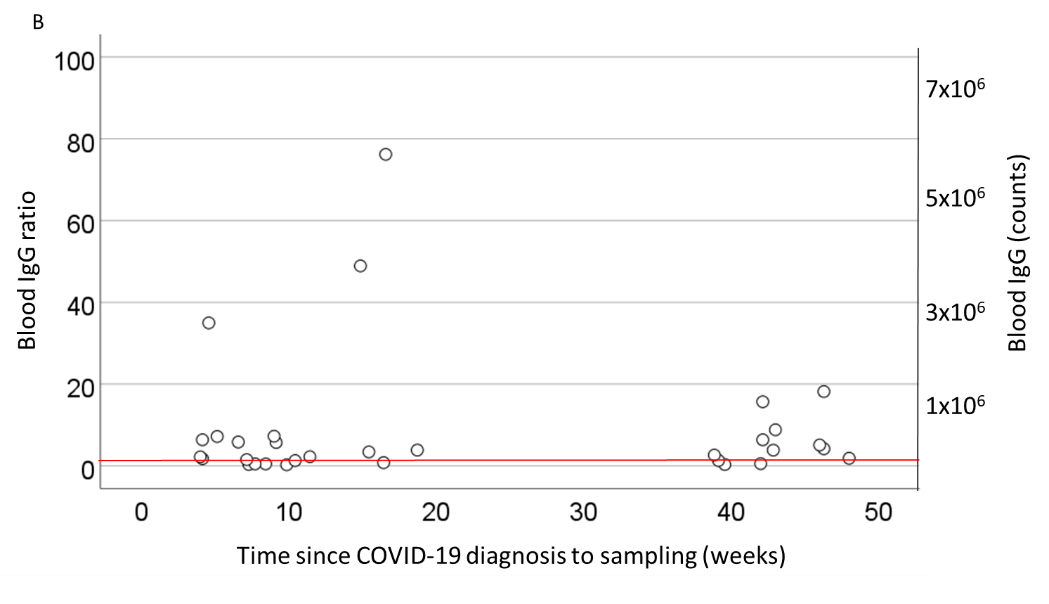


**Supplementary figure 3**. **Blood IgG after COVID-19 infection.** A) A total of 57 participants had had COVID-19 infection and were not vaccinated. B) 39 of those participants had presented a positive qPCR-test for COVID-19 infection. The figure shows the blood IgG ratio and counts. The red line is the cut-off limit for seropositivity.
